# Effects of acupuncture on gut microbiota and short-chain fatty acids in patients with functional constipation: a randomized placebo-controlled trial

**DOI:** 10.3389/fphar.2023.1223742

**Published:** 2023-09-01

**Authors:** Xiang-Yun Yan, Jun-Peng Yao, Yan-Qiu Li, Xian-Jun Xiao, Wan-Qing Yang, Si-Jue Chen, Tai-Chun Tang, Yu-Qing Yang, Liu Qu, Yu-Jun Hou, Min Chen, Ying Li

**Affiliations:** ^1^ School of Acupuncture and Tuina, Chengdu University of Traditional Chinese Medicine, Chengdu, China; ^2^ School of Health Preservation and Rehabilitation, Chengdu University of Traditional Chinese Medicine, Chengdu, China; ^3^ School of Clinical Medicine, Chengdu University of Traditional Chinese Medicine, Chengdu, China; ^4^ Hospital of Chengdu University of Traditional Chinese Medicine, Chengdu, China

**Keywords:** acupuncture, functional constipation, bioinformatic analysis, gut microbiota, SCFAs

## Abstract

**Objective:** To comprehensively evaluate the effect of acupuncture on gut microbiota, identify specific microbes closely related to the clinical efficacy of acupuncture, and explored the role of short-chain fatty acids (SCFAs).

**Methods:** A randomized placebo-controlled trial was conducted with 80 FC patients and 28 healthy controls (HCs). FC patients randomly received 16 acupuncture (*n* = 40) or sham acupuncture (*n* = 40) sessions over 4 weeks; HCs received no treatment. The change in the proportion of patients with mean weekly complete spontaneous bowel movements (CSBMs) was considered as the primary outcome measure. Moreover, the composition and the predictive metabolic function of the gut microbiota from feceal samples were analyzed by 16S rRNA gene sequencing, while feceal SCFAs were identified via gas chromatography-mass spectrometry (GC-MS).

**Results:** Compared to sham acupuncture, acupuncture significantly increased the proportion of CSBM responders, and improved spontaneous bowel movements (SBMs), straining, stool consistency, and quality of life. Moreover, Sequencing of 16S rRNA genes revealed that acupuncture improved β-diversity and restored the composition of gut microbiota. Specifically, the abundance of beneficial bacteria such as *g_Lactobacillus* increased while that of pathogenic bacteria such as *g_Pseudomonas* decreased after acupuncture, which were significantly correlated with alleviated symptoms. Moreover, ten microbes including *g_Coprobacter, g_Lactobacillus,* and *g_Eubacterium_coprostanoligenes_group* might be considered acupuncture-specific microbes, and formed a stable interaction network. Additionally, GC-MS analysis indicated that acupuncture increased the content of butyrate acid in the gut, which was positively correlated with an increase in defecation frequency and a decrease in acupuncture-related pathogens. Finally, acupuncture specific-microbes including *g_Coprobacter*, *g_Lactobacillus*, *g_Pseudomonas*, *g_Eubacterium_coprostanoligenes_group*, *g_Erysipelotrichaceae_UCG.003*, *g_Prevotellaceae_UCG.001*, and *g_Rolstonia* could accurately predict the clinical efficacy of acupuncture (AUC = 0.918).

**Conclusion:** Acupuncture could effectively improve clinical symptoms in FC patients, and was associated with gut microbiota reshaping and increased butyrate acid levels. Moreover, key microbial genera such as *g_Coprobacter* and *g_Lactobacillus* was predictive of acupuncture efficacy in treating FC. Future studies are required to validate the causal relationship between key microbial genera and acupuncture clinical efficacy, and should explore further metabolic pathways for designing personalized treatment strategies.

**Clinical Trial Registration:** http://www.chictr.org.cn, Identifier: ChiCTR2100048831.

## Graphical Abstract

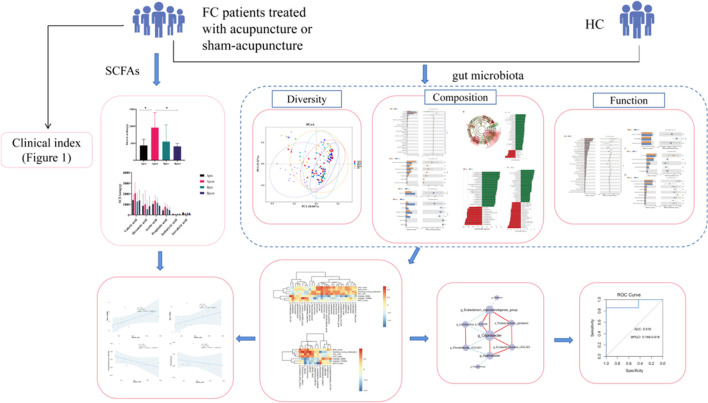

## Introduction

Functional constipation (FC) is a prevalent gastrointestinal disorder characterized by infrequent bowel movements, hard stools, and straining during defecation (Camilleri et al., 2017). FC can be considered a global health concern due to its high prevalence, low cure rate, and mental and economic burden (Dennison et al., 2005; Barberio et al., 2021). Although pharmacologic therapies such as laxatives and prokinetics serve as the current main management of FC, such therapies provide only temporary relief of symptoms, are costly when used for long periods, and have high relapse rates after withdrawal (Gordon et al., 2016). Furthermore, dissatisfaction with current constipation management is prevalent among approximately 50% of FC patients, leading them to seek complementary therapies with better efficacy and safety (Basilisco, 2020).

Acupuncture has been widely used to treat FC due to the rapid onset of perceived amelioration of symptoms and its steady long-term efficacy (Yao et al., 2022a). Recent studies have reported that acupuncture at acupoints Tianshu (ST25), Shangjuxu (ST37), and Fujie (SP14) is safe and effective in treating constipation (Liu et al., 2016). In fact, the efficacy of acupuncture was found to be comparable to that of prucalopride, the guideline-recommended drug for constipation treatment (Liu et al., 2021). A growing body of evidence has suggested that acupuncture may alleviate constipation by promoting intestinal motility (Shen et al., 2022), improving the function of the enteric nervous system (Liang et al., 2016), and regulating brain-gut interaction (Torres-Rosas et al., 2014). However, the underlying mechanism by which acupuncture can alleviate FC remains elusive.

In recent years, it has been reported that gut microbiota is involved in multiple pathological links to FC, i.e., slowed intestinal motility (Pan et al., 2022), low-grade intestinal inflammation (Gobert et al., 2016), impaired intestinal barrier function (Fu et al., 2021), and reduced intestinal mucosal secretion (Lu et al., 2021). Moreover, it has been described that metabolism is one of the most important functions attributed to the gut microbiota, and it has been suggested that its metabolic ability exceeds that of cells of the human body (Cani, 2019). Gut microorganisms can produce bioactive substances via their metabolic pathways, thereby restoring intestinal function. For instance, tryptophan is a metabolite that activates epithelial G protein-coupled receptors to increase colonic secretion (Bhattarai et al., 2018). In this context, short-chain fatty acids (SCFAs), the most well-studied and abundant metabolites of gut microorganisms, are formed from the metabolism of amino acids and pyruvate and promote the release of 5-HT to accelerate intestinal peristalsis (Akiba et al., 2015). However, the role of the gut microbiota and its related metabolic pathways in acupuncture treatment of FC patients is unclear.

In a previous study conducted by our research group, electroacupuncture was shown to increase α-diversity of gut microorganisms and decrease the *Firmicutes*/*Bacteroidetes* ratio in FC mice, thereby suggesting that electroacupuncture may promote gastrointestinal motility and alleviate constipation (Xu et al., 2021). However, α-diversity results were not in accordance with the observations of several reports on FC patients (Camilleri et al., 2017). Moreover, the previous study focused only on the overall structure of the gut microbiota, and did not explore changes at the genus level and/or its metabolic functions. Therefore, it is necessary to comprehensively demonstrate the effects of acupuncture on the gut microbiota and its metabolic pathways in human subjects.

Consequently, we conducted a randomized placebo-controlled trial recruiting patients with FC and healthy volunteers to comprehensively evaluate the effect of acupuncture on gut microbiota, identify the specific microbes closely related to the efficacy of acupuncture, and explore the role of SCFAs. The findings in the present study provide a theoretical basis for explaining the mechanisms by which acupuncture partly alleviated the symptoms of FC, which points towards an individualized clinical application of acupuncture.

## Materials and methods

### Study design

**FIGURE 1:**
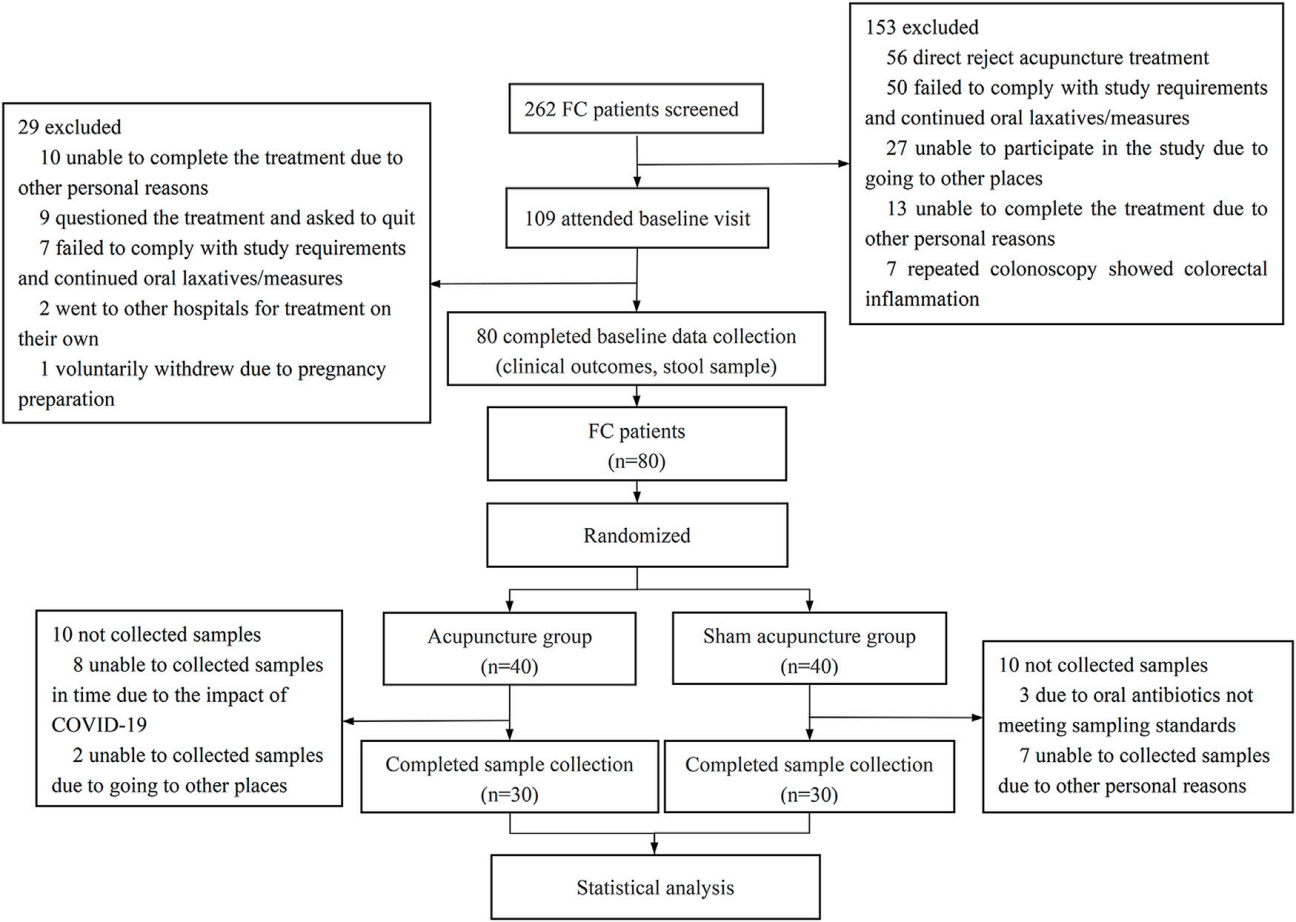
Flow chart of clinical research.

We conducted a randomized, sham-controlled trial from 1 April 2021, to 1 October 2022. A total of 262 patients were enrolled through the Chengdu University of Traditional Chinese Medicine Affiliated Hospital Outpatient Department and the WeChat platform. After the subjects signed the informed consent form, a baseline observation of 1 week was conducted, during which patients ceased laxative use and maintained normal diets. 80 functional constipation patients were randomized into acupuncture (*n* = 40) or sham-acupuncture (*n* = 40) groups using SPSS 26.0 (Figure 1). Cubicles separated subjects to prevent communication. Inclusion and exclusion criteria are detailed in Supplementary Table S1. Additionally, 28 healthy volunteers served as controls, providing baseline stool samples only. The 4-week treatment phase involved acupuncture or sham-acupuncture sessions. Local ethics committee approval (Sichuan Provincial Commission of Traditional Chinese Medicine Regional Ethics Review, Approval No. 2021KL-023) and registration with the China Clinical Trial Registry (ChiCTR2100048831) were obtained.

### Intervention

During the 4-week study, participants received 16 treatment sessions, with 5 times a week for the first 2 weeks and 3 times a week for the next 2 weeks. Treatments were performed by a licensed acupuncturist with at least 3 years of experience in functional constipation treatment. The acupuncture group received stimulation at bilateral *ST25*, *SP14*, and *ST37* acupoints, while the sham group received stimulation at three non-acupuncture points using a *Park Sham Device (PSD)*. The acupuncture group received vertical needle insertions (0.30 × 40 mm or 0.30 × 50 mm) to produce pain, distension, numbness, or heaviness sensations. The sham group received non-penetrating stimulation with a retractable placebo needle (*Hwato* brand, size 0.30 × 40 mm). Both groups had 30-min sessions and the acupuncture group received needle insertion every 10 min, while the sham group did not. Acupuncture points, non-acupuncture points, and placebo needles for the sham group are detailed in Supplementary Figures S1, S2; Supplementary Table S2. PSD use ensured participant and investigator blinding to treatment assignment.

### Clinical outcomes

Participants were required to complete a stool diary for the duration of the 4-week study period and complete the Patient Assessment of Constipation Quality of Life questionnaire (PAC-QOL), the Self-Rating Anxiety Scale (SAS) score, and the Self-Rating Depression Scale (SDS) score at both baseline and week 4. The stool diary comprised several key components, including mean weekly spontaneous bowel movements (SBMs), mean weekly complete spontaneous bowel movements (CSBMs), stool consistency, and straining. The primary outcome measure was changed in the proportion of patients with mean weekly CSBMs. Secondary outcome measures included changes in mean CSBMs, SBMs, Bristol Stool Form Scale (BSFS) scores (ranging from 1 to 7 for stool types 1 to 7, respectively), straining scores (scores of 0–3 indicating increasing levels of difficulty defecating), PAC-QOL, SAS and SDS scores.

### Collection and quality control of fecal samples

Stool samples from 80 patients with FC will be collected at baseline and post-treatment, while 28 healthy volunteers will provide baseline samples only. Participants will maintain their regular diet, avoiding antibiotics and probiotic products, such as yogurt, for 7 days before collection. They will also refrain from strenuous exercise, coffee, strong tea, and other stimulants 24 h before collection. Women should wait until after menstruation to provide samples. Participants will receive comprehensive instructions for stool collection using the *Fecal Microbial Genome Protection Solution Kit* (Guangdong Nanxin Medical Technology Co., Ltd.). Two fecal tubes will be collected for gut microbiome and SCFA analysis. Samples can be collected at home or the hospital, per participant preference. Approximately 3 g of clean, formed stool from the middle section should be collected and immediately stored in a −80°C freezer at the hospital or temporarily in a 4°C refrigerator for up to 2 h. Samples must be transferred to the research staff for gut microbiome and SCFA analysis within 24 h.

Despite not restricting gender during recruitment, our study ended up with only one male functional constipation patient and no male healthy volunteers due to a higher prevalence of constipation in women and potential sociocultural influences. We believe this distribution does not impact our research on acupuncture’s effects on gut microbiota in functional constipation patients, as our primary comparison lies between the acupuncture and sham acupuncture groups, not between genders.

### Fecal sample DNA extraction and 16S rRNA gene sequencing

To prepare samples for sequencing, total genomic DNA was extracted using the CTAB method and checked for concentration and purity with 1% agarose gel. DNA was diluted to 1 ng/μL using sterile water. Next, specific regions of the *16S rRNA/18SrRNA/ITS* genes (such as *16S V4/16S V3/16S V3-V4/16S V4-V5, 18S V4/18S V9, ITS1/ITS2, Arc V4*) were amplified using specific primers with a barcode.

PCR reactions utilized Phusion^®^ High-Fidelity PCR Master Mix and 10 ng of template DNA, followed by thermal cycling and 2% agarose gel detection. PCR products were purified using a Qiagen Gel Extraction Kit, and sequencing libraries were generated with the TruSeq^®^ DNA PCR-Free Sample Preparation Kit, adding index codes. Library quality was assessed using a Qubit@ 2.0 Fluorometer and an Agilent Bioanalyzer 2,100 system. Sequencing was performed on an Illumina NovaSeq platform, producing 250 bp paired-end reads.

### Microbial analysis

Sequencing data were analyzed using Uparse and DADA2 module or blur in QIIME2 software to reduce noise and filter out sequences with an abundance below 5, yielding ASVs and a feature table. The classify-skarn module in QIIME2 was used to obtain species information by comparing ASVs to a database. Alpha diversity was assessed using four indices (Chao1, Shannon, Simpson, Pielou_e) calculated with QIIME2. Beta diversity was compared using weighted and unweighted UniFrac distances and visualized with Principal Coordinates Analysis (PCoA) in R software (Version 3.5.3). A petal plot was drawn to show ASV number differences and a relative abundance accumulation map depicted bacterial abundance variations across groups. The *t*-test and linear discriminant analysis effect size (LEfSe) were used to identify significant abundance differences among groups (biomarkers), with a linear discriminant analysis (LDA) score threshold of 2.5. Specific acupuncture-effect microorganisms were preliminarily screened using Venn analysis (https://bioinfogp.cnb.csic.es/tools/venny/index.html).

### Correlation and co-occurrence analysis

The correlation between acupuncture or sham-acupuncture-specific microbes and clinical indicators were displayed using heat maps with Spearman analysis. Furthermore, We make a correlation interaction co-occurrence network diagram of the bacteria related to the clinical effect of acupuncture.

### PICRUSt analysis

Functional prediction of the gut microbiome was performed using the Phylogenetic Investigation of Communities by Reconstruction of Unobserved States test (PICRUSt) based on the Kyoto Encyclopedia of Genes and Genomes (KEGG) orthology. Stamp analysis was used to find the function with significant differences between the two groups assessed using the *t*-test.

### Fecal SCFAs analysis

Prepare eight mixed standard concentration gradients of acetic, propionic, butyric, isobutyric, valeric, isovaleric, and caproic acids using ethyl acetate. Add 25 μL internal standard solution (500 μM 4-methyl valeric acid) to 600 μL standard solution and centrifuged samples. Mix, centrifuge, and use 150 μL supernatant for analysis. Analyze samples using an Agilent 7890A/5975C GC-MS on an Agilent DB-WAX capillary column (30 m × 0.25 mm ID × 0.25 μm). Calculate concentrations of six SCFAs based on peak areas. Linear plot of the correlation between butyric acid and clinical index, acupuncture-specific microbes were displayed using Spearman correlation analysis.

### Random forest model

We conducted random forest analysis based on microbes’ abundance using R software (Version 3.5.3) and selected different numbers of microbes to build a random forest model. Through two indicators Accuracy and Specificity out of important species, and draw the receiver operating characteristic (ROC) curve.

### Statistical analysis

We determined our sample size based on a proportion difference Z-test. With an anticipated effect size of 32% derived from previous studies, a desired statistical power of 80% (*β* = 0.2), and a Type I error rate of 5% (*α* = 0.05), our calculations yielded a required total sample size of 80 participants for this study.

Normally distributed data are presented as mean ± standard deviation values, while non-normally distributed data are shown as median and interquartile range. The chi-square test in SPSS (version 23.0) was employed for changes in the proportion of average weekly CSBM patients, and the *t*-test was utilized to evaluate efficacy differences between and within groups. Flora analysis was conducted using R software. A *p*-value of <0.05 was considered statistically significant.

## Results

### Consistency in baseline characteristics across groups

80 FC patients and 28 healthy controls who met the inclusion criteria were included in the study. There was no statistically significant difference in sex, age, and BMI index between FC patients and HCs (all *p* > 0.05). The acupuncture and sham-acupuncture groups showed no significant difference in sex, age, BMI, and any clinical characteristics (Table 1).

**TABLE 1:** Characteristics of participants in different groups and healthy controls.

Characteristic	Acupuncture group (*n* = 40)	Sham-acupuncture group (*n* = 40)	*p*-value	FC (*n* = 80)	HC (*n* = 28)	*p*-value
Sex (female) n (%)	39 (97.50)	39 (97.50)	0.753	78 (97.50%)	28 (100%)	0.398
Age, M (IQR)	27.50 (15.50)	27.00 (9.50)	0.497	27.00 (10.75)	26.50 (8.25)	0.113
BMI, mean (SD)	20.68 ± 2.29	20.67 ± 2.25	0.980	20.68 ± 2.26	21.47 ± 2.35	0.185
Mean constipation duration, M (IQR), mo	120.00 (98.25)	108.00 (144.00)	0.261	—	—	—
Mean CSBMs per week, M (IQR), n	0.50 (1.00)	1.00 (2.00)	0.126	—	—	—
Mean SBMs per week, M (IQR), n	3.00 (1.00)	3.00 (0.75)	0.158	—	—	—
Mean bristol stool form scale score (BSFS) for stool consistency of SBMs, mean (SD)	2.85 ± 1.27	2.92 ± 0.80	0.753	—	—	—
Mean straining score of SBMs, M (IQR)	1.00 (0.67)	1.00 (0.32)	0.330	—	—	—
Mean PAC-QOL score, mean (SD)	2.51 ± 0.66	2.52 ± 0.68	0.970	—	—	—
Mean SAS score, M (IQR)	38.75 (11.25)	41.25 (8.13)	0.478	—	—	—
Mean SDS score, mean (SD)	41.72 ± 9.04	44.88 ± 9.75	0.137	—	—	—

### Acupuncture improved clinical symptoms of patients with FC

Both groups exhibited improvements in SBMs, BSFS, straining defecation, PAC-QOL, SAS, and SDS scores compared to baseline (*p* < 0.05), but sham-acupuncture had no significant effect on CSBMs (*p* > 0.05) (Supplementary Table S3). The proportion of patients with CSBMs ≥3 times per week was 75% for the acupuncture group, significantly higher than the 7.50% for the sham-acupuncture group (*p* < 0.001). Additionally, the changes in mean weekly SBMs (*p* = 0.003), BSFS (*p* = 0.040), straining defecation (*p* = 0.005), and PAC-QOL (*p* = 0.004) were statistically different between the acupuncture and sham-acupuncture groups. However, no significant differences were observed in mean weekly SAS and SDS scores between the two groups after intervention (*p* = 0.084, *p* = 0.325, respectively) (Table 2).

**TABLE 2:** Comparison of changes in clinical variables after treatment of 4 weeks between two groups.

Outcome	Acupuncture group (*n* = 40)	Sham-acupuncture group (*n* = 40)	*p*-value
Primary outcome			
The proportion of patients with mean weekly CSBMs ≥3, %	75.00	7.50	<0.001
Secondary outcomes (post-pre) (95% CI)			
Change of mean weekly CSBMs, mean (SD)	3.59 ± 2.03 (2.94, 4.24)	0.60 ± 1.33 (0.18, 1.02)	<0.001
Change of mean weekly SBMs, mean (SD)	3.45 ± 1.90 (2.84, 4.06)	2.27 ± 1.38 (1.83, 2.71)	0.003
Change of mean weekly straining during defecation, mean (SD)	−0.81 ± 0.55 (−0.99, −0.63)	−0.43 ± 0.53 (−0.60, −0.26)	0.005
Change of mean weekly BSFS, mean (SD)	1.11 ± 1.05 (0.77, 1.45)	0.73 ± 0.79 (0.48, 0.98)	0.040
Change of PAC-QOL score, mean (SD)	−1.07 ± 0.56 (−1.25, −0.90)	−0.67 ± 0.66 (−0.88, −0.45)	0.004
Change of SAS score, M (SD)	−9.13 ± 6.96 (−11.35, −6.90)	−5.34 ± 8.13 (−7.94, −2.74)	0.084
Change of SDS score, mean (SD)	−7.88 ± 6.58 (−9.98, −5.77)	−6.09 ± 9.31 (−9.07, −3.11)	0.325

### Acupuncture modulated the diversity of the gut microbiome

**FIGURE 2:**
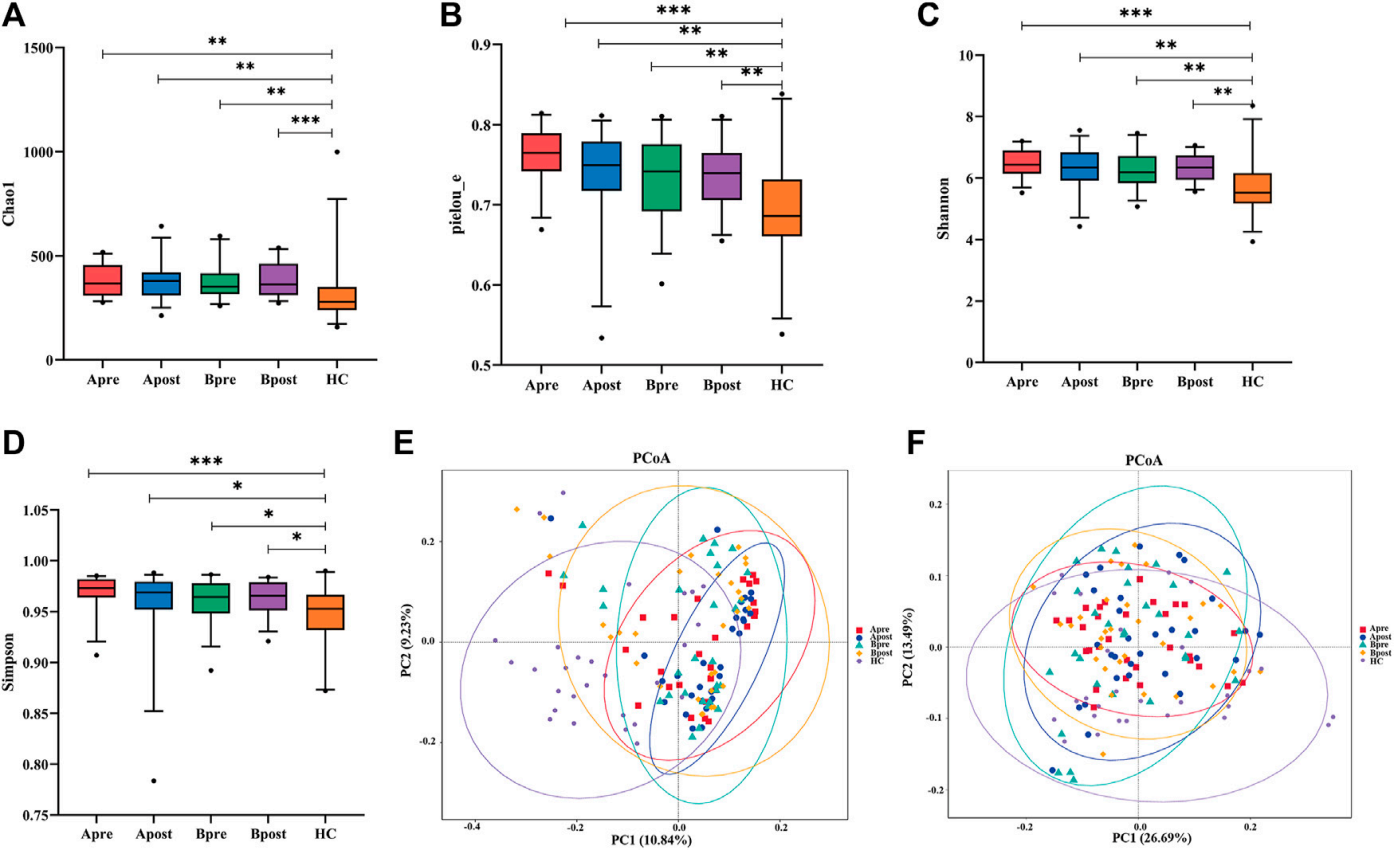
Comparison of gut microbial diversity. **(A–E)** α-diversity of gut microbiota among the Apre group (*n* = 30), the Apost group (*n* = 30), the Bpre group (*n* = 30), the Bpost group (*n* = 30), and the HC group (*n* = 28) based on Chao1, Pielou_e, Simpson, Shannon, and dominant indices (Wilcoxon rank-sum test, *p < 0.05, **p < 0.01, ***p < 0.001). **(F)** Unweighted-Unifrac-based principal coordinate analysis (PCoA in Adonis) between healthy controls, acupuncture group, or sham-acupuncture group pre- and post-treatment (HC vs. Apre or Bpre, *p* = 0.001, Apre vs. Apost, *p* = 0.017, Apost vs. Bpost, *p* = 0.01). Apre, before acupuncture treatment; Apost, after acupuncture treatment; Bpre, before sham acupuncture treatment; Bpost, after sham acupuncture treatment; HC, healthy controls.

The study results indicate that sequencing depth and sample size are adequate, as shown by the stabilization of the rarefaction curve and species accumulation boxplot (Supplementary Figure S3). Alpha diversity indices, including Chao1, Pielou’s evenness, Shannon, and Simpson, represent community richness, evenness, and diversity. In comparison to HC, patients with FC showed a significant increase in all four indices (*p* < 0.05). However, the Alpha diversity of gut microbiota did not show significant changes in either the acupuncture or sham-acupuncture groups (*p* > 0.05) (Figures 2A–D).

β-diversity analysis was conducted to compare the gut microbial communities of different groups. PCoA plots were generated using weighted and unweighted UniFrac distances to show samples with similar gut microbial compositions appearing closer together (Figures 2E, F). Results showed that the weighted UniFrac distance of PCoA analysis did not indicate any statistical difference between groups, while the unweighted UniFrac distance demonstrated that the gut microbial communities of the HC group are distinct from those of FC patients (Adonis test, *R* ^2^ = 0.97, *p* = 0.001). After the acupuncture treatment, the gut microbial communities of FC patients diverge from those before acupuncture (Adonis test, *R* ^2^ = 0.97, *p* = 0.017) and become more similar to those of the HC group. However, gut microbial communities after sham-acupuncture intervention do not show significant differences from those before treatment (*p* > 0.05).

### Acupuncture restored the composition of the gut microbiome

**FIGURE 3:**
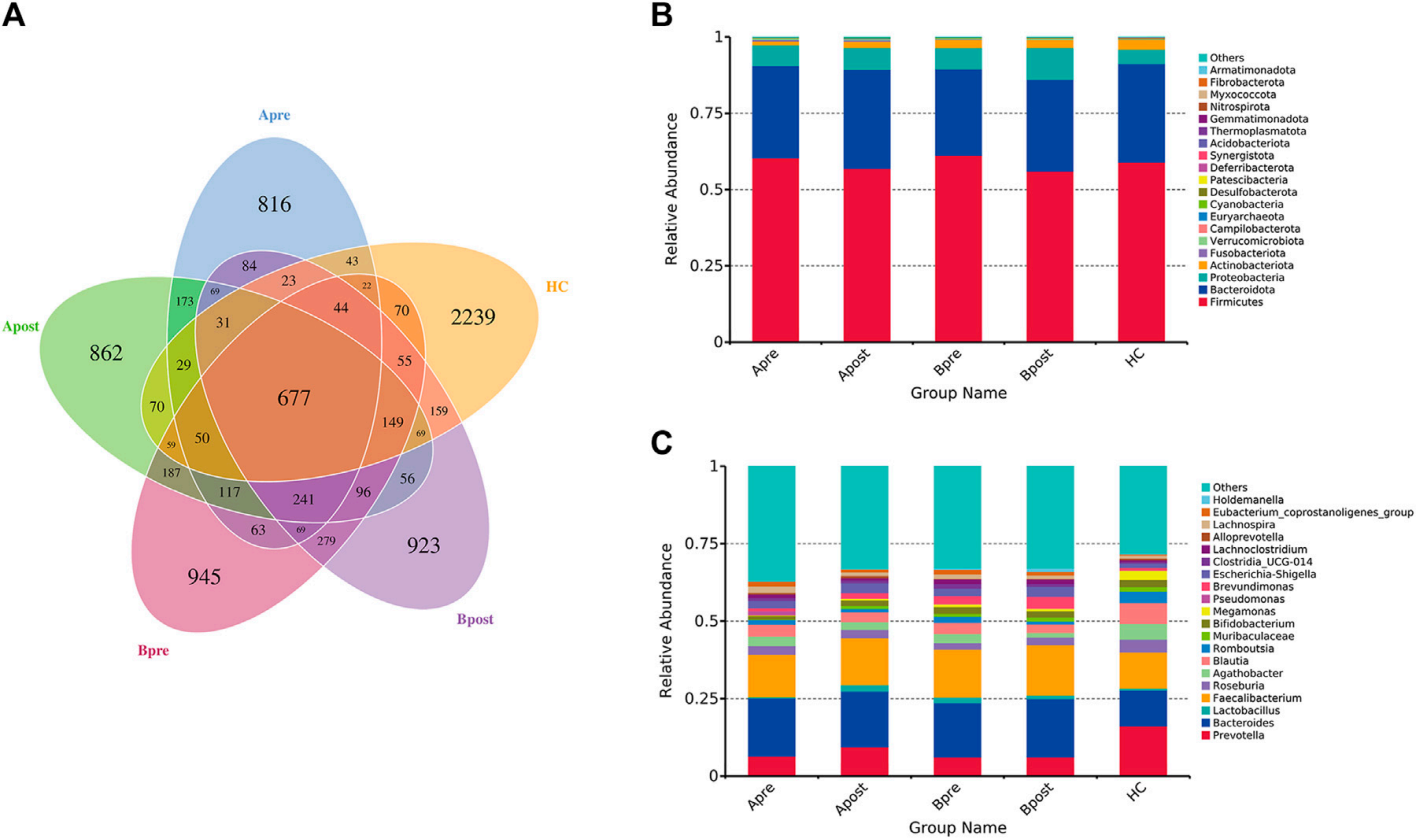
The change in the number of ASV and the abundance of intestinal flora. **(A)** Petal diagram of ASV number of intestinal flora in five groups **(B)** Stacked histogram of relative abundance of taxa at the phylum level **(C)** Stacked histogram of relative abundance of taxa at the genus level. Apre, before acupuncture treatment; Apost, after acupuncture treatment; Bpre, before sham acupuncture treatment; Bpost, after sham acupuncture treatment; HC, healthy controls.

A petal diagram (Figure 3A) displayed the changes in ASVs between groups. The HC group had 2,239 unique ASVs, while patients had 816 before acupuncture treatment, increasing to 862 after acupuncture treatment. Conversely, patients had 945 unique ASVs before sham-acupuncture treatment, decreasing to 923 after sham-acupuncture. The stacked bar chart showed the top 20 phyla and genera. At the phylum level, *Firmicutes*, *Bacteroidetes*, *Proteobacteria*, and *Actinobacteria* had the highest proportions (Figure 3B). At the genus level, *Prevotella*, *Bacteroidetes*, and *Faecalibacterium* were enriched in all five groups. Several beneficial species, such as *Bifidobacterium*, and *Lactobacillus* increased, while other potentially harmful species, such as *Pseudomonas* decreased after acupuncture (Figure 3C). After sham-acupuncture, the relative abundance of beneficial bacteria *Lactobacillus* was even reduced. These findings suggest that acupuncture can better regulate the balance of microbial composition than sham-acupuncture.

**FIGURE 4:**
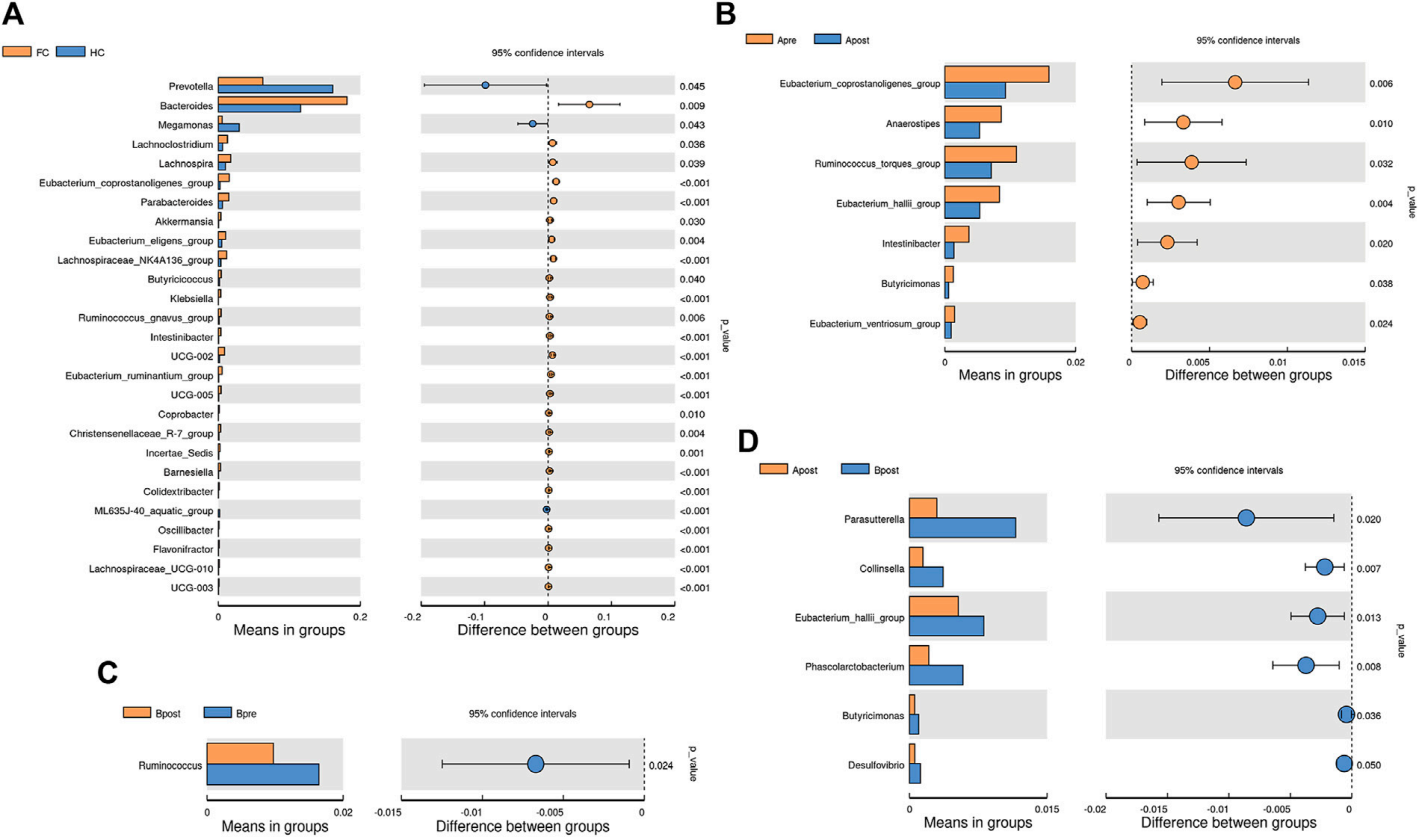
Differential bacteria between groups based on *t*-test. The bar plot of the difference genus between the FC and healthy controls **(A)**, between the Apre and Apost group **(B)**, or between the Bpre and Bpost group **(C)**, or between the Apost and Bpost group **(D)**. (T-test, *p* < 0.05). Apre, before acupuncture treatment; Apost, after acupuncture treatment; Bpre, before sham acupuncture treatment; Bpost, after sham acupuncture treatment; HC, healthy controls.

To identify microbes with significant differences between the groups, a *t*-test was carried out. Compared to HC, patients with FC showed a significant increase in the abundance of 24 genera (Figure 4A). After acupuncture treatment, the abundance of 7 genera decreased, including *g_Eubacterium_coprostanoligenes_group* (*p* = 0.006) and *g_Anaerostipes* (*p* = 0.010) (Figure 4B). However, in the sham-acupuncture group, only one genus, *g_Ruminococcus* (*p* = 0.024) showed a significant decrease (Figure 4C). Moreover, the abundance of 6 genera including *g_Parasutterella* (*p* = 0.020), *g_Phascolarctobacterium* (*p* = 0.008) significantly decreased after acupuncture treatment compared to sham-acupuncture treatment (Figure 4D).

**FIGURE 5:**
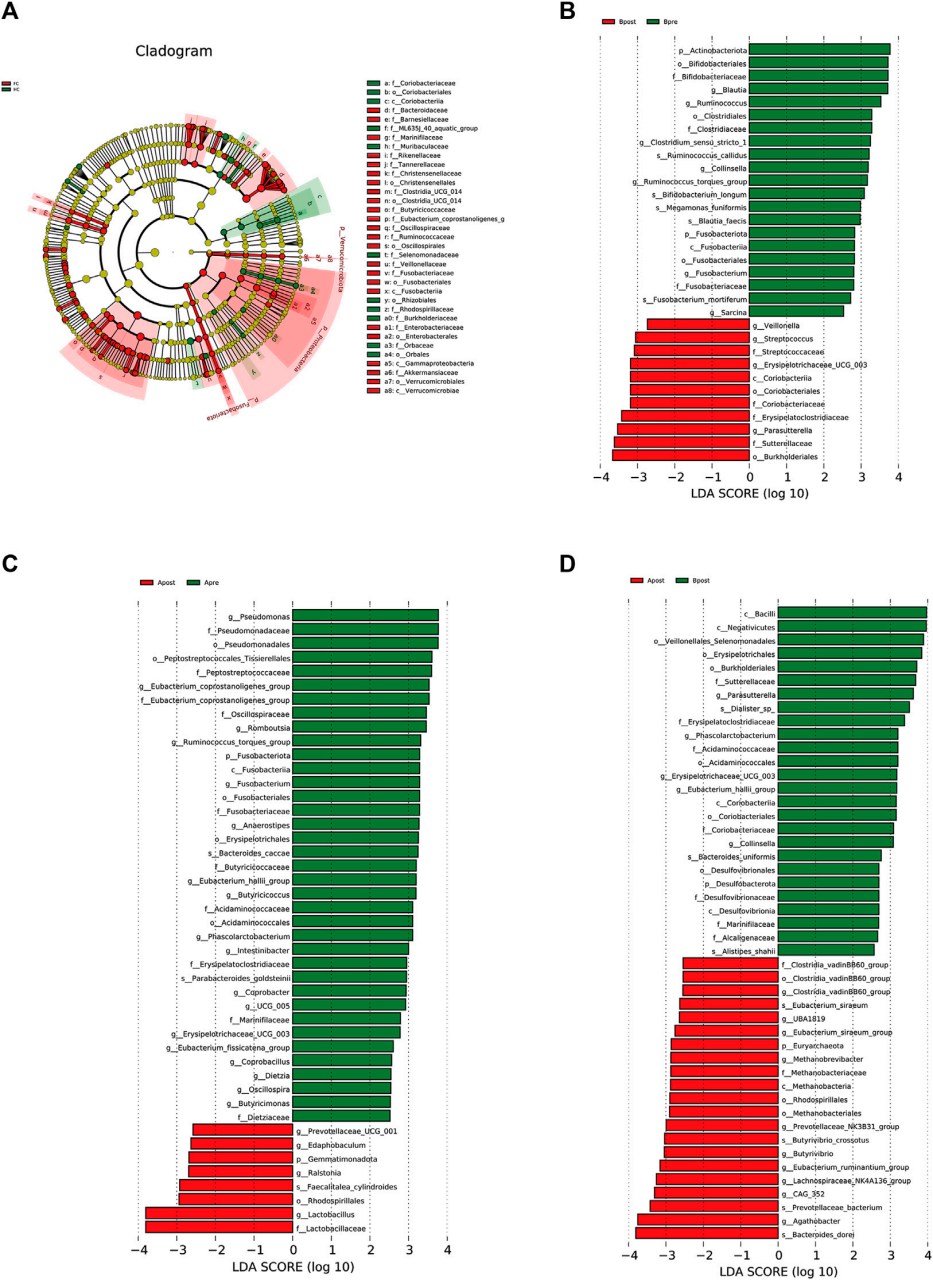
LEfSe analysis of 16S rRNA-based microbiota abundance **(A)** Evolutionary branch diagram of the differential intestinal flora of FC patients and healthy subjects (LDA >2.5, *p* < 0.05). **(B)** The taxonomy bar chart of the differential intestinal flora before and after sham-acupuncture (*n* = 30) (LDA >2.5, *p* < 0.05). **(C)** The taxonomy bar chart of the differential intestinal flora before and after acupuncture (*n* = 30) (LDA >2.5, *p* < 0.05). **(D)** The taxonomy bar chart of the differential intestinal flora after acupuncture and sham-acupuncture (*n* = 30) (LDA >2.5, *p* < 0.05). Apre, before acupuncture treatment; Apost, after acupuncture treatment; Bpre, before sham acupuncture treatment; Bpost, after sham acupuncture treatment; HC, healthy controls.

To explain the differences between groups, we used LEfSe analysis, which takes into the phylogenetic relatedness between taxa. We visualized the LEfSe results with an LDA score >2.5. The results showed that *p_Proteobacteria* and *p_Fusobacteriota* were more abundant in the FC group than the HC group (Figure 5A). After acupuncture treatment, the abundance of *g_Lactobacillus* increased, while that of *g_Pseudomonas*, *f_Peptostreptococcaceae*, and *g_Eubacterium_corprostanoligenes_group* decreased (Figure 5C). In the sham-acupuncture group, the abundance of *o_Burkholderriales*, *f_Sutterellaceae* increased while that of *g_Blautia*, *p_Actinobacteriota* decreased (Figure 5B). Additionally, *s_Bacteroides_dore*, *g_Agathobacter,* and *s_Prevotellaceae_bacterium* were enriched more in the acupuncture group than the sham-acupuncture group (Figure 5D).

### Preliminary screening of acupuncture specific-microbes

To preliminarily screen for key intestinal bacterial genera/species for acupuncture treatment of FC, we used Venn’s logistic analysis. First, we merged the genera and species of differences before and after acupuncture treatment and between groups, and excluded microorganisms with relative abundances less than 0.0001 in each group or 90% of the samples (Supplementary Tables S4, S5).

Next, we removed microbes whose abundances changed in the same direction after acupuncture treatment, after sham-acupuncture treatment, and in the FC group (Supplementary Figure S4). The results showed that *g_Collinsella* decreased among the three groups, while *g_Ruminococcus_torques_group* and *g_Fusobacterium* decreased after both acupuncture and sham-acupuncture treatment. Meanwhile, 9 microbes increased both in FC patients and after acupuncture treatment. Finally, 12 microbes were eliminated and 28 significantly different species of acupuncture-specific regulation were screened out.

### Acupuncture specific-microbes were associated with clinical index

Spearman correlation analysis was performed to evaluate the relationships between clinical indices and gut microbiota in the acupuncture and sham-acupuncture groups.

**FIGURE 6:**
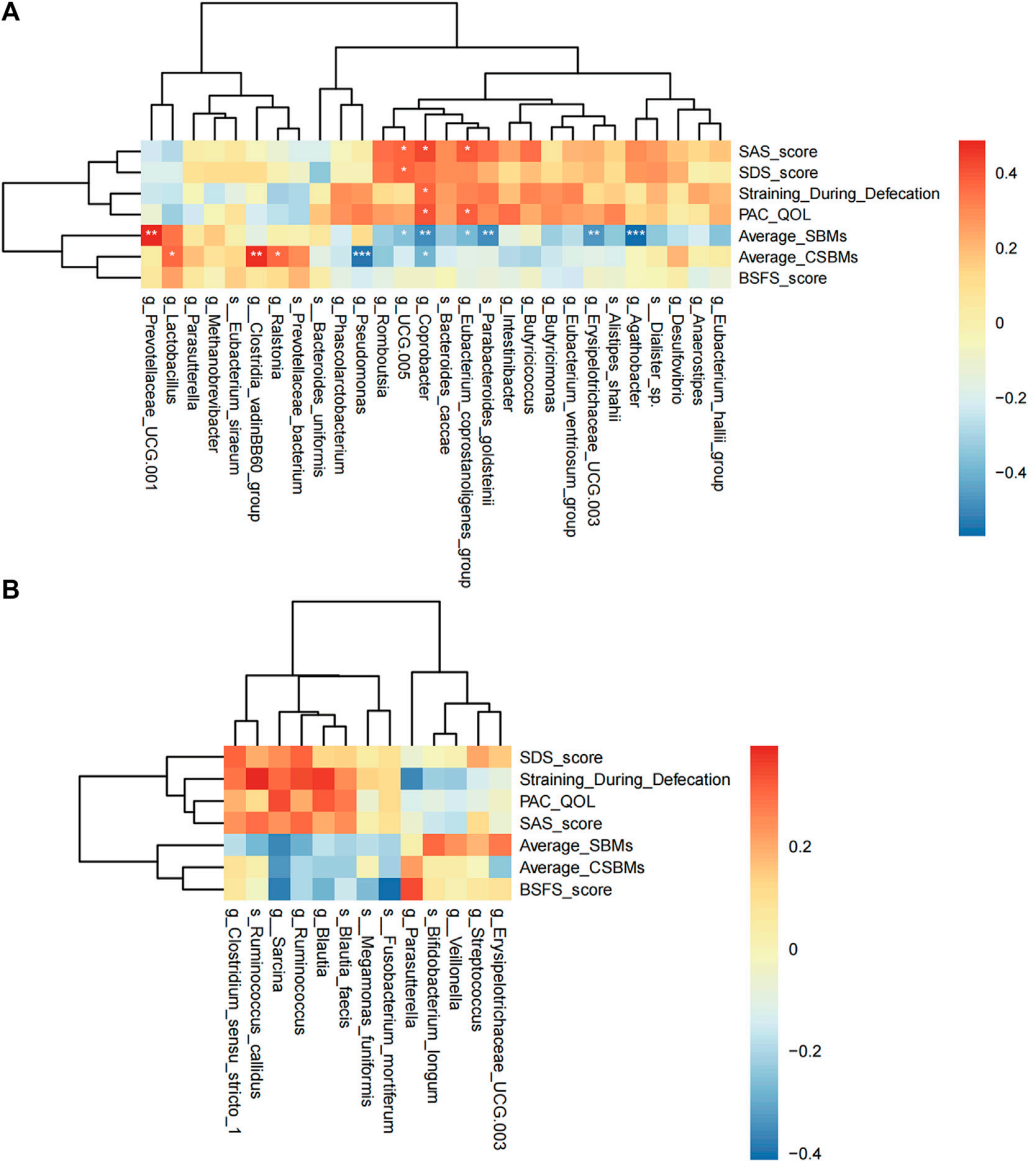
Spearman correlation analysis. The correlation heat map of the acupuncture **(A)** or sham-acupuncture **(B)** specific-microbes and clinical indicators. The color depth represents the strength of correlation, red represents positive correlation, and blue represents negative correlation. *p*-value corrected by Benjamin Hochberg, *q < 0.05, **q < 0.01, ***q < 0.001.

In the acupuncture group, there are 11 microbes significantly related to clinical indicators (Figure 6A). *g_Coprobacter* was not only significantly negatively correlated with CSBMs (*r* = −0.399, *q* = 0.029) and SBMs (*r* = −0.487, *q* = 0.003), but positively correlated with straining defecation (*r* = 0.373, *q* = 0.043), PAC-QOL score (*r* = 0.401, *q* = 0.029), and SAS score (*r* = 0.431, *q* = 0.014). *g_Lactobacillus* (*r* = 0.367, *q* = 0.043), *g_Clostridia_vadinBB60_group* (*r* = 0.472, *q* = 0.004), and *g_Ralstonia* (*r* = 0.368, *q* = 0.043) were positively correlated with the mean weekly CSBMs, while *g_Pseudomonas* (*r* = −0.537, *q* < 0.001) was significantly negatively correlated with. However, in the sham-acupuncture group (Figure 6B), there were no microbes related to clinical indicators.

### Acupuncture specific-microbes formed an interaction co-occurrence network

**FIGURE 7:**
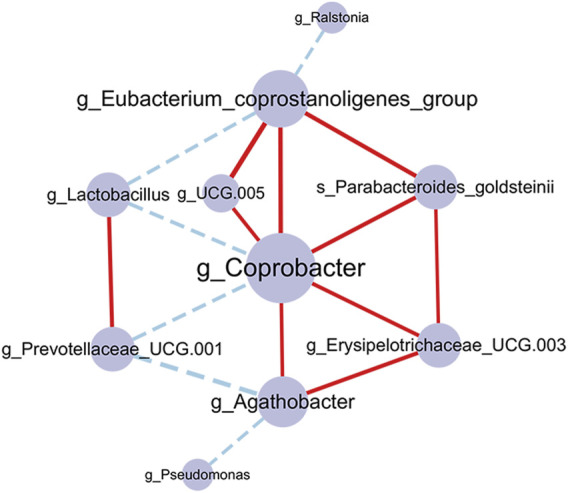
Co-occurrence network of the gut microbiome in the acupuncture group. The larger the node and label, indicating the greater the centrality. The edges represent correlations between microbes, the red connection is a positive correlation, and the blue connection is a negative correlation. The thickness of the edge indicates the strength of the correlation. *p*-value corrected by Benjamin Hochberg.

We also constructed a correlation network to illustrate the community relationships among acupuncture related-microbes. There were 10 nodes and 16 edges in the network and the density of the network was 0.178. The average degree centrality of the network is 3. In the network (Figure 7), *g_Coprobacter* had the highest centrality (degree = 7), which was positively correlated with 5 microbes including *g_Eubacterium_coprostanoligenes_group* (*r* = 0.455, *p* = 0.002), and *g_Erysipelotrichaceae_UCG.003* (*r* = 0.376, *p* = 0.012), while negatively correlated with *g_Lactobacillus* (*r* = −0.372, *p* = 0.012) and *g_Prevotellaceae_UCG.001* (*r* = −0.362, *p* = 0.015). In addition, there are more positively correlated connections among bacteria in the network than negatively correlated, indicating the relationship between bacteria is mainly synergistic. These 10 interacted bacteria maintain the stability of the gut microbiome and play an important role in the acupuncture treatment of FC.

### Function predictor of gut microbiota controlled by acupuncture treatment

**FIGURE 8:**
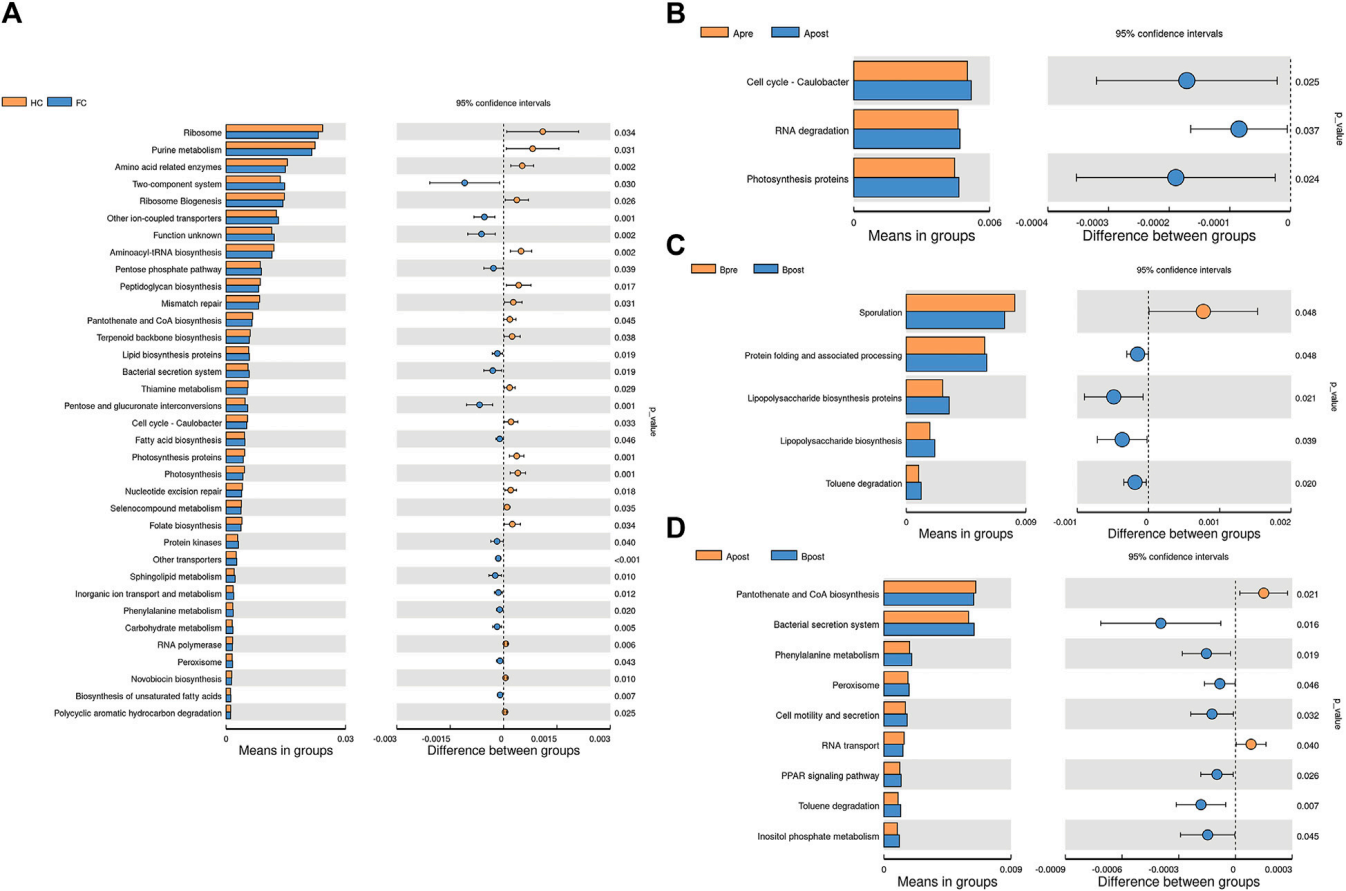
Picrust-predicted flora functions differed between groups. Differences in microbiota function between HC and FC patients **(A)**, between the Apre and Apost group **(B)**, between the Bpre and Bpost group **(C)**, or between the Apost and Bpost group **(D)**. Apre, before acupuncture treatment; Apost, after acupuncture treatment; Bpre, before sham acupuncture treatment; Bpost, after sham acupuncture treatment; HC, Healthy controls.

To elucidate the function of the microbiota associated with acupuncture treatment for FC patients, we compared the functional KEGG pathway enrichment predicted by PICRUSt analysis. A total of 34 pathways were at level 3 and many of the predicted functions were in metabolic pathways. For example, purine metabolism, amino acid-related enzymes, and Pantothenate and CoA biosynthesis were significantly decreased in the FC group (*p* < 0.05) (Figure 8A). After acupuncture treatment, the level of metabolism, such as photosynthesis proteins, D-Alanine metabolism, and Isoflavonoid biosynthesis increased significantly (*p* < 0.05) (Figure 8B; Supplementary Table S6). However, after sham-acupuncture, the relative abundance of Lipopolysaccharide biosynthesis, and Toluene degradation increased (*p* < 0.05) (Figure 8C). Acupuncture, when compared to sham-acupuncture, led to an increase in Pantothenate and CoA biosynthesis and a decrease in the Phenylalanine metabolism (*p* < 0.05) (Figure 8D).

### Acupuncture regulated the content of fecal SCFAs

**FIGURE 9:**
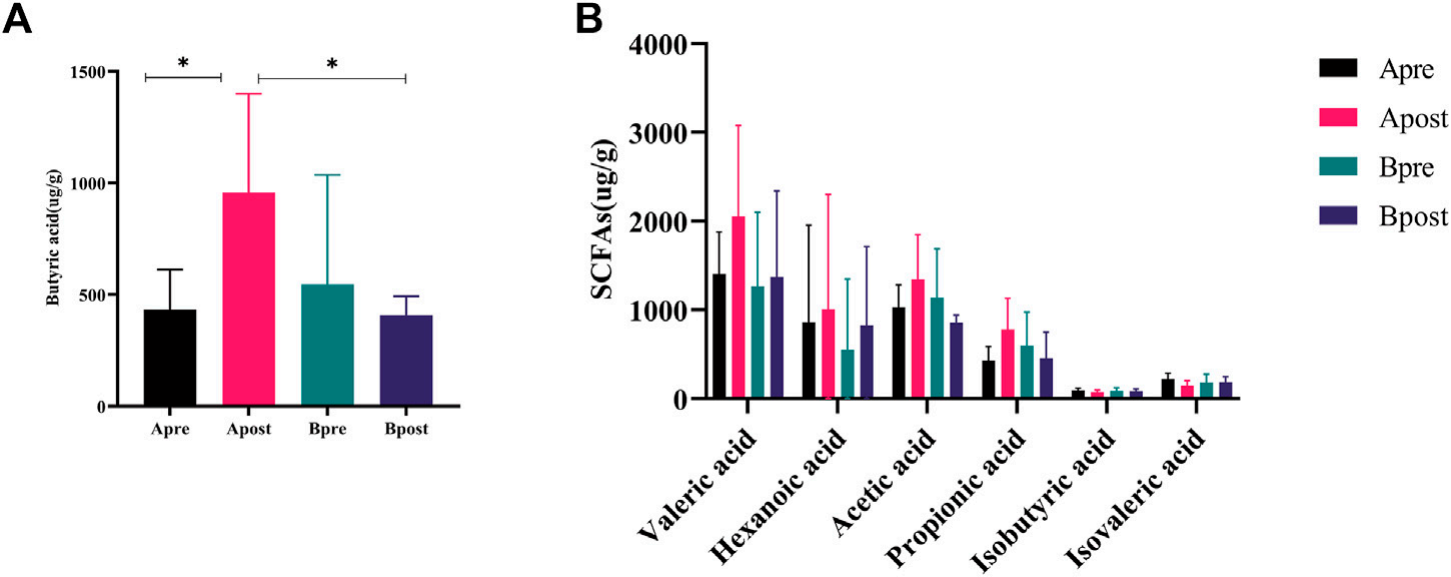
Fecal SCFAs of acupuncture and sham-acupuncture groups before and after treatment. **(A)** Change of the level of Butyric acid between the Apre group (*n* = 5), Apost group (*n* = 5), Bpre group (*n* = 5), and Bpost group (*n* = 5). *t*-test, *p < 0.05. **(B)** Change of the level of remaining SCFAs levels between the Apre group (*n* = 5), Apost group (*n* = 5), Bpre group (*n* = 5), and Bpost group (*n* = 5). Apre, before acupuncture treatment; Apost, after acupuncture treatment; Bpre, before sham acupuncture treatment; Bpost, after sham acupuncture treatment.

Fatty acid metabolism plays an important role in microbial function. To compare the changes in SCFAs content before and after treatment, 5 patients in each group were measured. Compared with baseline, the level of butyric acid in patients after acupuncture treatment increased (*p* < 0.05), and there was no significant change in SCFAs after sham-acupuncture treatment. Compared with the sham-acupuncture group, the level of butyric acid in the acupuncture group increased significantly after treatment (*p* < 0.05) (Figure 9A). However, there was no significant change in the other 6 SCFAs (Acetic acid, Propionic acid, Valeric acid, Hexanoic acid, Isovaleric acid, and Isobutyric acid) between any two groups (Figure 9B). Additionally, Spearman correlation analysis showed that butyric acid was positively correlated with the mean weekly CSBMs (*r* = 0.693, *p* = 0.026) and SBMs (*r* = 0.742, *p* = 0.014), while negatively correlated with *g_Erysipelotrichaceae_UCG.003* (*r* = −0.782, *p* = 0.012) and *g_Pseudomonas* (*r* = −0.649, *p* = 0.049) (Supplementary Figure S5).

### Gut microbiota-based prediction of acupuncture effect based on random forest model

**FIGURE 10:**
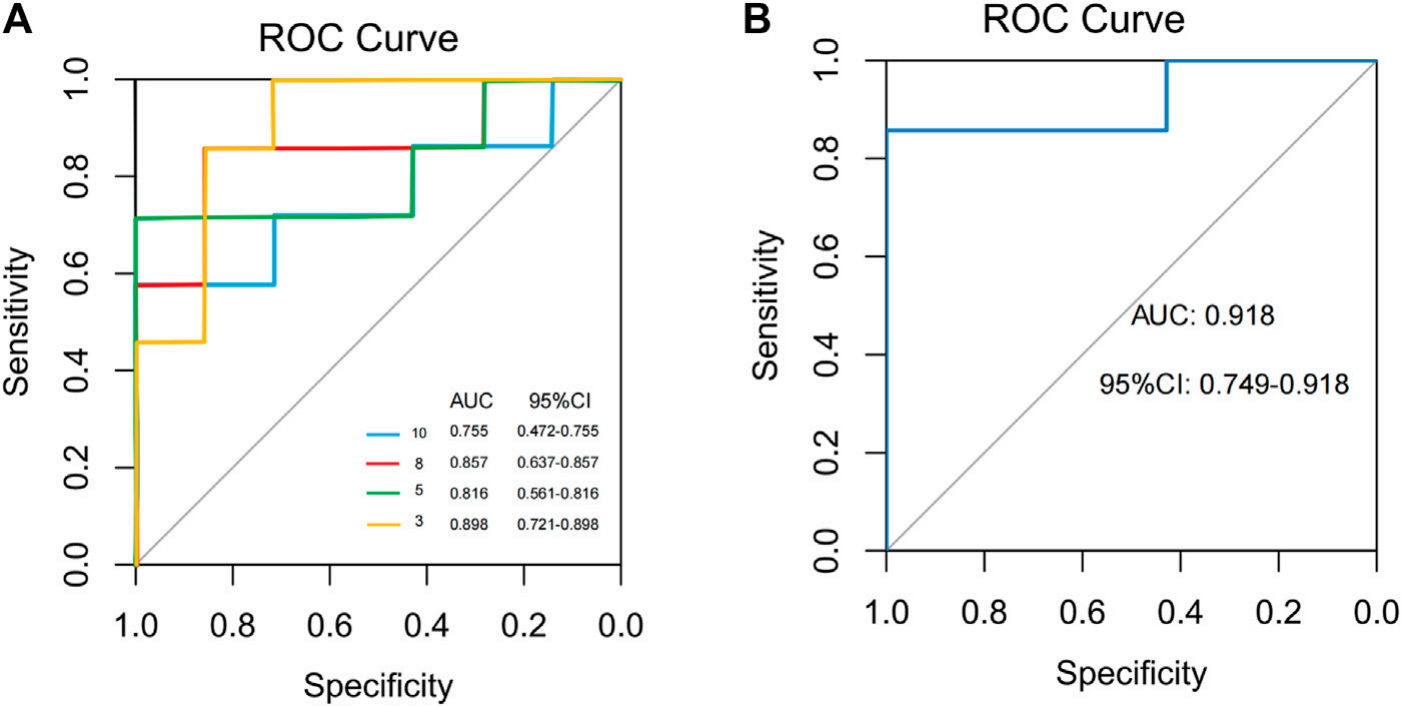
Classification performance of a random forest model using different microbes. **(A)** The random forest model used 3, 5, 8, and 10 different genera or species. **(B)** The random forest model used 7 different genera.

To find and test the potential gut microbiota biomarkers, a random forest model was applied based on 10 microbes from a co-occurrence network. After comprehensive consideration of the value of the AUC (Figures 10A, B), seven potential diagnostic gut microbiota biomarkers (*g_Coprobacter*, *g_Lactobacillus*, *g_Pseudomonas*, *g_Eubacterium_coprostanoligenes_group*, *g_Erysipelotrichaceae_UCG.003*, *g_Prevotellaceae_UCG.001*, *g_Rolstonia*) were screened out and were attempted to be constructed as a diagnostic model with AUC = 0.918.

## Discussion

Herein, we conducted a randomized trial to evaluate the impact of acupuncture on the gut microbiota and its metabolic function in FC patients, which enabled the identification of specific microbes related to the acupuncture intervention. This study revealed that acupuncture significantly improved the clinical symptoms of FC and restored the composition and function of the gut microbiota in FC patients. It was further revealed that the significant clinical efficacy of acupuncture was related to the increase in beneficial bacteria and the decrease in opportunistic pathogenic bacteria. Specific acupuncture-related microbial genera including *g_Lactobacillus* and *g_Coprobacter* were identified and could accurately predict the efficacy of acupuncture intervention to FC patients. In addition, acupuncture increased the content of butyric acid in the gut, which was associated with an increase in defecation frequency and a decrease in acupuncture-specific pathogens.

### Confirmation of the clinical effect of acupuncture at acupoints ST25, ST37, and SP14

It is well established that the primary physiopathologic mechanism of FC is decreased intestinal motility associated with reduced sensitivity of both sympathetic and parasympathetic nervous systems. Previous studies have demonstrated that acupuncture at acupoints *ST25*, *ST37*, and *SP14* can effectively increase bowel movements (Liu et al., 2016; Yao et al., 2022b). It has been described that the deep sections of *ST25* and *SP14* correspond to the nerve segments of the large intestine (T10) and the small intestine (T11), respecitevely, which exert bidirectional influence on peristalsis and contraction via sympathetic or parasympathetic nerves (Yu et al., 2016; Zeng and Chen, 2022).

In this study, acupuncture proved more effective than sham-acupuncture in increasing the proportion of patients with mean weekly CSBM ≥3, mean weekly CSBMs, SBMs, and ameliorating straining defecation, fecal dryness and quality of life (*p* < 0.05). Collectively, these findings indicate that acupuncture targeting the acupoints *ST25*, *ST37*, and *SP14* has a net therapeutic effect to FC, which are in accordance with the results of a previous study (Camilleri et al., 2017).

However, sham acupuncture may also improve constipation symptoms to a certain extent, which is consistent with previous studies (Menees et al., 2020). The results obtained herein may be attributed to the placebo effect of the intervention, which is more prominent in functional gastrointestinal diseases (Enck et al., 2017). Nevertheless, it was found that the sham acupuncture intervention did not result in changes in the primary outcome measure in FC patients (*p* > 0.05). It is known that the sensation of incomplete defecation is associated with intestinal mechano-sensing dysfunction, which suggests that the placebo effect did not affect FC-related mechano-sensing dysfunction.

### Acupuncture alters the composition of gut microbiota in patients with FC

The gut microbiota acts as a bridge role between intestinal health and acupuncture. In this study, it was found that acupuncture or sham acupuncture had no effect on α-diversity, although there was a tendency for α-diversity to decrease after acupuncture intervention, which is in contrast with the results of our previous experiments (Xu et al., 2021). Similarly, changes in α-diversity in the gut microbiota of FC patients are controversial. This study found that α-diversity was increased in FC patients compared to HC, while other studies reported lower α-diversity in FC patients (Zhang et al., 2021). These discrepancies may be due to variations in subject characteristics, such as gender and age (Li et al., 2021). Therefore, further studies are needed to elucidate changes in α-diversity of the gut microbiota in FC patients.

Moreover, it was observed that acupuncture reduced more significantly similarity in gut microbiota community structure than sham acupuncture. Notably, β-diversity analysis using both weighted and unweighted methods yielded inconsistent results, which may be related to the fact that, contrarily to unweighted methods, weighted methods consider the relative abundance of species. These observations suggest that species whose relative abundance changed after acupuncture may be important in shaping community structure and function.

Furthermore, and consistent with the results of previous studies (Xu et al., 2021), the observations of the present study revealed that acupuncture could lead to a significant increase in the relative abundance of beneficial microbial genera (e.g., *g_Lactobacillus*) and a decrease in the abundance of potentially harmful microbial genera (e.g., *g_Pseudomonas*). Moreover, it was found that sham acupuncture decreased the relative abundance of beneficial bacteria in the gut of FC patients, thus worsening dysbiosis. Taken together, it was shown herein that, compared to sham acupuncture, acupuncture was more effective in restoring and rebalancing gut microbiota structure.

### Assessing the possible role of acupuncture-related microbes in the treatment of FC

After screening for differential microbes, specific microbes associated with acupuncture-related changes in the gut microbiota were evaluated both at the genus and species level. Specifically, *g_Lactobacillus* and *g_Prevotellaceae_UCG.001* were found increased after acupuncture and positively correlated with CSBMs and SBMs, respectively. *g_Lactobacillus* can promote the production of various biologically active substances, such as metabolites, serotonin, SCF/c-kit protein secretion, etc., thereby enhancing intestinal peristalsis (Chen et al., 2023). Meanwhile, *g_Lactobacillus* has antibacterial activity, can inhibit pathogenic bacteria, reduce intestinal inflammatory response and protect intestinal epithelial cells and mucous membranes (Eor et al., 2019). Additionally, *g_Prevotellaceae_UCG.001* could promote intestinal peristalsis by SCFAs production, thereby increasing the water content in feces due to the degradation of mucus proteins and by changing ion transmembrane transport in the intestinal lumen (Yuan et al., 2022). Acupuncture may modulate the intestinal environment in multiple ways, such as reducing oxidative stress (Zhang et al., 2022), regulating immunity, reducing inflammation (Yang et al., 2021), and thereby increase the abundance of *g_Lactobacillus* and *g_Prevotellaceae_UCG.001*. Therefore, both of them are beneficial microbes and can be used as probiotics or prebiotics to prevent or treat intestinal functional disorders such as constipation and inflammatory bowel disease. Moreover, they can also act as synergistic factors for acupuncture treatment, improving the safety and tolerance of acupuncture treatment.Among bacteria whose relative abundance was downregulated after acupuncture treatment, *g_Coprobacter* was significantly correlated with all clinical efficacy indicators except for BSFS and SDS. In addition, *g_Eubacterium_coprostanoligenes_group* was negatively correlated with SBMs while positively correlated with PAC-QOL. *g_Coprobacter* and *g_Erysipelotrichaceae_UCG.003* can cause damage to intestinal barrier function and weaken intestinal absorption by producing endotoxin and affecting bile acid metabolism; it can also inhibit the growth of beneficial bacteria by competing for nutrients or releasing antibacterial substances, thereby disrupting the balance and balance of intestinal microorganisms (Tian et al., 2020; Sheh et al., 2022). *g_Eubacter_coprostanoligenes_group* increases the excretion and conversion of 7α-dehydroxylase, affects the production, secretion or circulation of bile acids, and then disturbs the secretion and regulation of intestinal hormones, resulting in weakened intestinal absorption, intestinal peristalsis and defecation dysfunction (Guo et al., 2022). *g_Pseudomonas* damages the intestinal barrier function by releasing endotoxin, extracellular DNA and extracellular enzymes, leading to increased intestinal permeability and inflammatory response; at the same time, it inhibits the growth of beneficial bacteria, increases the drug resistance and tolerance of bacteria, disrupt gut homeostasis (Liu et al., 2020). Acupuncture can inhibit the production of inflammatory factors (Bao et al., 2022), and the secretion of gastric acid (Takahashi, 2006), reducing the colonization and inhibiting the toxicity of pathogenic microbes. Therefore, these microbes can be used as biomarkers for diagnosing or monitoring intestinal dysfunction, as well as evaluation indicators for acupuncture treatment, so as to optimize acupuncture programs and improve acupuncture effects.

Additionally, the observations further revealed that the effect of acupuncture on FC could not be attributed to a single bacterial species but rather to the synergistic and antagonistic interactions among multiple bacterial species which form a stable bacterial community structure and maintain intestinal homeostasis. Specifically, synergic interaction among gut bacteria is related to metabolite sharing (Culp and Goodman, 2023) or information exchange through signaling molecules, thus maintaining a more stable and resilient microbiota (Oliveira et al., 2023). Conversely, antagonistic interactions encompass competition for resources, e.g., *Lactobacillus* producing antibacterial molecules to suppress the invation of pathogenic bacteria (van Zyl et al., 2020).

Finally, further studies should be conducted to explore the interactions among these microbial genera to elucidate the holistic and regulatory effects of acupuncture in FC patients.

### Acupuncture regulated gut microbiota metabolic pathways in FC patients

Microorganisms can participate in various metabolic pathways, such as the metabolism of carbohydrates, proteins and lipids. They can also decompose and transform the food components ingested by the host to produce various metabolites, such as short-chain fatty acids, amino acid metabolites, vitamins, etc (Moszak et al., 2020). Different microbial communities will produce different metabolite profiles (Nicholson et al., 2012). These differences may affect the utilization of nutrients and the production of metabolites by gut microbes, resulting in metabolic differences among groups.

It is widely accepted that the active metabolic pathways in microbes in the gut play an important role in maintaining intestinal health. Herein, it was found that the metabolic pathways photosynthesis, D-alanine metabolism, and isoflavonoid biosynthesis were increased in FC patients after acupuncture, which likely affected the growth of gut microbes. For instance, D-alanine metabolism is involved in peptidoglycan synthesis and biofilm formation, thereby contributing to maintaining the integrity of the bacterial cell wall (Wei et al., 2016). In addition, it has been shown that isoflavonoid biosynthesis can promote the growth of certain beneficial bacterial species such as *Bifidobacteria* and restore intestinal health (Gaya et al., 2020). Collectively, these results indicate that acupuncture may have affected the regulation of the afore-mentioned metabolic pathways in gut microbes, which in turn may affect the growth of certain bacterial species and restore the balance of the gut microbiota.

SCFAs are important metabolic end products that play a critical role in intestinal health by mediating the anti-inflammatory response, increasing colonic smooth muscle contractility, and regulating intestinal neurotransmission (He et al., 2020). In particular, butyric acid can promote the secretion of lysozyme and defensin by Paneth cells, repair damaged intestinal mucosa (Takakuwa et al., 2019), and promote the release of 5-HT to restore intestinal motility (Vincent et al., 2018). Herein, it was found that the content of butyrate acid increased in FC patients after acupuncture treatment, which was positively correlated with an increase in defecation frequency and negatively correlated with a decrease in pathogens. It can be speculated the increase in butyric acid content after acupuncture may relieve constipation symptoms by regulating the release of neurotransmitters and hormones and by inhibiting the growth of undesirable bacteria. However, it should be noted that butyric acid has a bidirectional regulatory effect on intestinal motility, promoting intestinal motility at low doses but inhibiting it at high doses (Parthasarathy et al., 2016), which may partly explain the two-way effect of acupuncture in FC patients. Furthermore, other SCFAs did not change significantly after acupuncture, suggesting that factors such as diet and exercise may also influence the levels of these SCFAs (Seethaler et al., 2022; Liang et al., 2023). Therefore, further research is needed to fully elucidate the effect of acupuncture on the regulation of microbial metabolic pathways in the gut of FC patients.

### Clinical application of specific microbes regulated by acupuncture

Several studies have demonstrated the potential of gut microbiota function prediction in personalized nutrition development (Zmora et al., 2019), disease diagnosis and monitoring (Bosch et al., 2022), and drug response and development (Haiser et al., 2013). Herein, it was further found that seven bacterial genera, i.e., *g_Coprobacter*, *g_Lactobacillus*, *g_Pseudomonas*, *g_Eubacterium_coprostanoligenes_group*, *g_Erysipelotrichaceae_UCG.003*, *g_Prevotellaceae_UCG.001*, and *g_Rolstonia*, had high predictive power for the positive effect of acupuncture in alleviating FC symptoms (AUC = 0.918). Since these microbial indicators are not affected by the expressive ability of the patients, their language system and cognitive function (Yiannakou et al., 2015), which may be considered more accurate and objective indexes for evaluating the clinical efficacy of acupuncture. Also, these microbial indicators can assist clinicians in predicting and monitoring acupuncture efficacy prior to treatment of FC. Moreover, they can be used to guide personalized treatment plans, such as determining the most suitable acupoints and treatment duration for the needs of the patient, thereby maximizing treatment effect.

### Limitations of the present study

Firstly, although the present study included a small sample size, especially for the number of samples used for short-chain fatty acid (SCFA) analysis, which may result in higher variability, the quality of samples analyzed herein was strictly controlled. Moreover, the placebo effect was excluded to ensure the reliability of the obtained data to the greatest extent. Secondly, it was not possible to elucidate the causal relationship between FC and gut microbiota. In future studies, fecal microbiota transplantation or a “sterile” model will be used to elucidate the causal relationship observed in the present study. Third, due to the influence of the epidemiological characteristics of constipation, the proportion of male patients in this study is low, and future research can increase the proportion of male samples to explore the impact of gender on acupuncture efficacy and intestinal flora.

In conclusion, this study reported that acupuncture can significantly improve the clinical symptoms in patients with FC, which is associated with altered gut microbiota and butyrate acid levels. Acupuncture specific-microbes, such as *Lactobacillus* and *Coprobacter* may accurately predict the acupuncture effect before recieving treatment. Future studies should validate the causal relationship of these genera and clinical efficacy of acupuncture and explore more metabolic pathways for personalized treatment strategies.

## Data Availability

The original contributions presented in the study are publicly available. This data can be found here: https://figshare.com/, 10.6084/m9.figshare.23744736.
