# Supplementary material for: Effects of acupuncture on gut microbiota and short-chain fatty acids in patients with functional constipation: a randomized placebo-controlled trial
Source: Front Pharmacol. 2023 Sep 1;14:1223742. doi: 10.3389/fphar.2023.1223742 (PMC10502303; doi:10.3389/fphar.2023.1223742)
Supplement: Supplementary file 1 [file DataSheet1.docx]

***Supplementary Material***

**Effect of Acupuncture on Gut Microbiota and Short-chain Fatty Acids in Patients with Functional Constipation: A Randomized Placebo-Controlled Trial**

Xiang-Yun Yan^1†^, MM; Jun-Peng Yao^1†^, MD; Yan-Qiu Li^1†^, MM; Xian-Jun Xiao^3†^, MD; Wan-Qing Yang^2^, MM; Si-Jue Chen^1^, MM; Tai-Chun Tang^2^, MM; Yu-Qing Yang^1^, MD; Liu Qu^1^, MM; Yu-Jun Hou^1^, MM; Min Chen^2,*^, MD, PhD.; Ying Li^1,*^, MD, PhD

1 **Supplementary Data**

**Supplementary Figure S1**. Location of acupoints or sham acupoints in the trial.

Location of acupoints or sham acupoints in the trial. ST25, Tianshu; SP14, Fujie; ST37, Shangjuxu. NA, non-acupoint.

**Supplementary Figure S2**. Schematic diagram of a placebol needle device.

**Supplementary Figure S3**. The rarefaction curve and species accumulation curve analysis of bacterial 16S rRNA.

The rarefaction curve is the process of randomly selecting a certain amount of sequencing data from a sample, counting the number of species they represent (i.e. OTUs), and constructing a curve based on the amount of sequencing data extracted and the corresponding number of species. The curve can directly reflect the rationality of the sequencing data volume and indirectly reflect the richness of species in the sample. When the curve tends to be flat, it indicates that the sequencing data volume is gradually reasonable. The abscissa of the species accumulation boxplot represents the sample size and the vertical axis represents the number of OTUs. If the boxplot position shows a sharp increase, it indicates that a large number of species have been discovered in the community; When the position of the boxplot tends to be flat, it indicates sufficient sampling and allows for data analysis.

**Supplementary Figure S4**. Venn diagram of species differences between groups based on the same direction of action.

Venn diagram of species differences between groups based on the same direction of action. Venne analysis of microbes whose relative abundance increased (A) or decreased (B) after acupuncture treatment, after sham-acupuncture treatment, and in FC patients.

**Supplementary Figure S5**. Spearman correlation between butyric acid and clinical index, acupuncture-related-specific microbes.

Spearman correlation between butyric acid and clinical index, acupuncture-related specific microbes. Linear plot of the correlation between butyric acid and mean_CSBMs (A), mean_SBMs (B), g_Erysipelotrichaceae_UCG.003 (C), g_Pseudomonas (D) before (n = 5) and after acupuncture (n = 5).

**Supplementary Table S1**. The inclusion and exclusion criteria for FC patients

**Supplementary Table S2**. Acupoints and non-acupoints used in the acupuncture group

**Supplementary Table S3**. Comparison of clinical variables at the baseline and the end of the study in different groups.

**Supplementary Table S4**. Specific changes in bacterial genera/species after acupuncture treatment.

**Supplementary Table S5**. Specific changes in bacterial genera/species after sham acupuncture treatment.

**Supplementary Table S6**. Predictive function of gut microbiota in differential changes after acupuncture treatment.

2 **Supplementary Figures and Tables**

Supplementary Figure S1

| 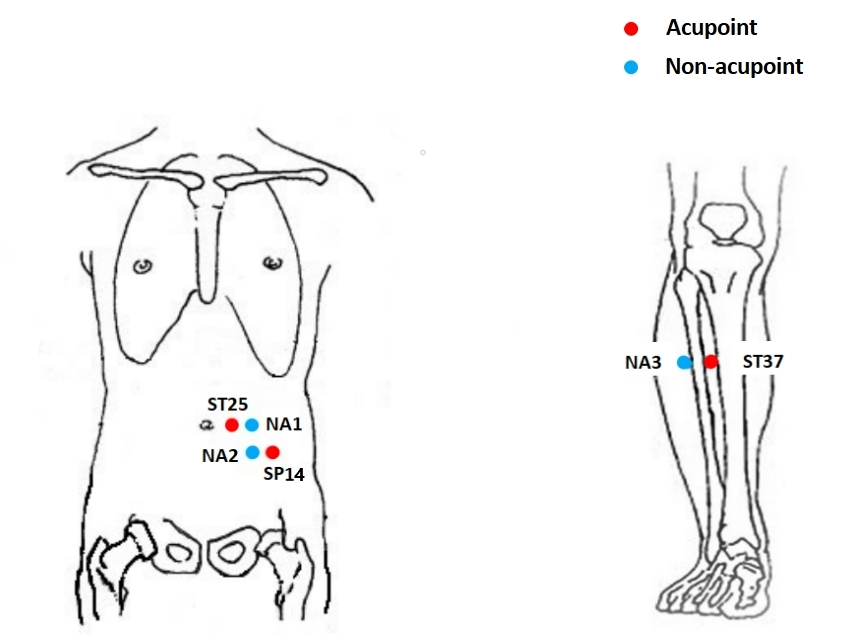 |
| --- |

Supplementary Figure S2

| 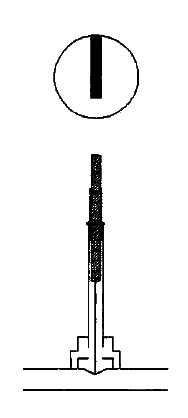 |
| --- |

Supplementary Figure S3

| 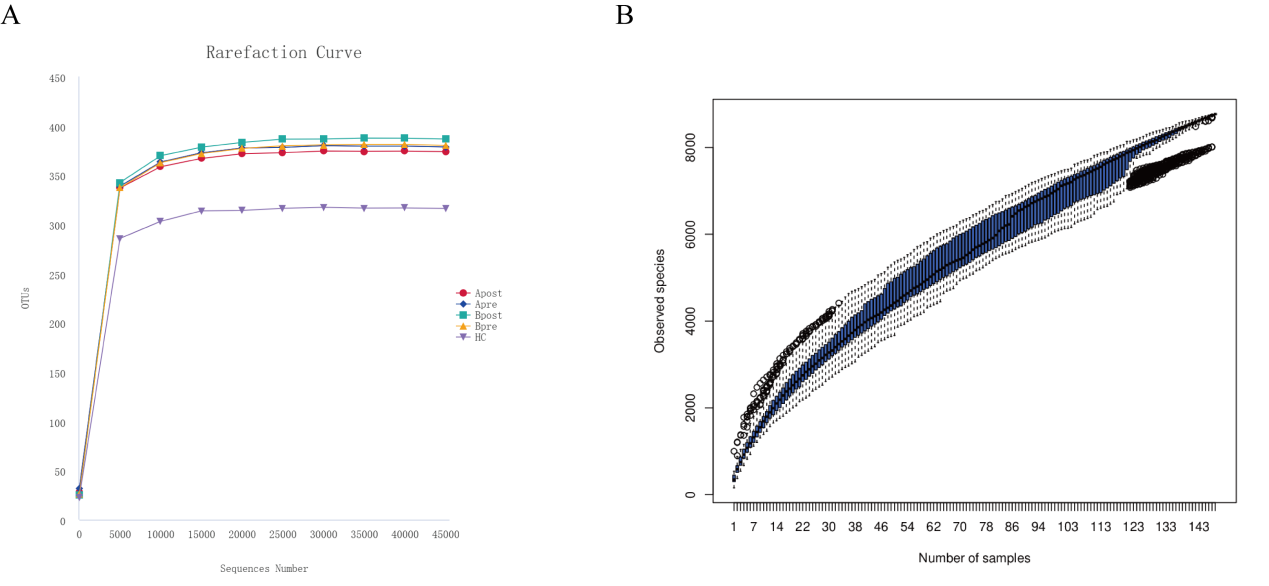 |
| --- |

Supplementary Figure S4.

| 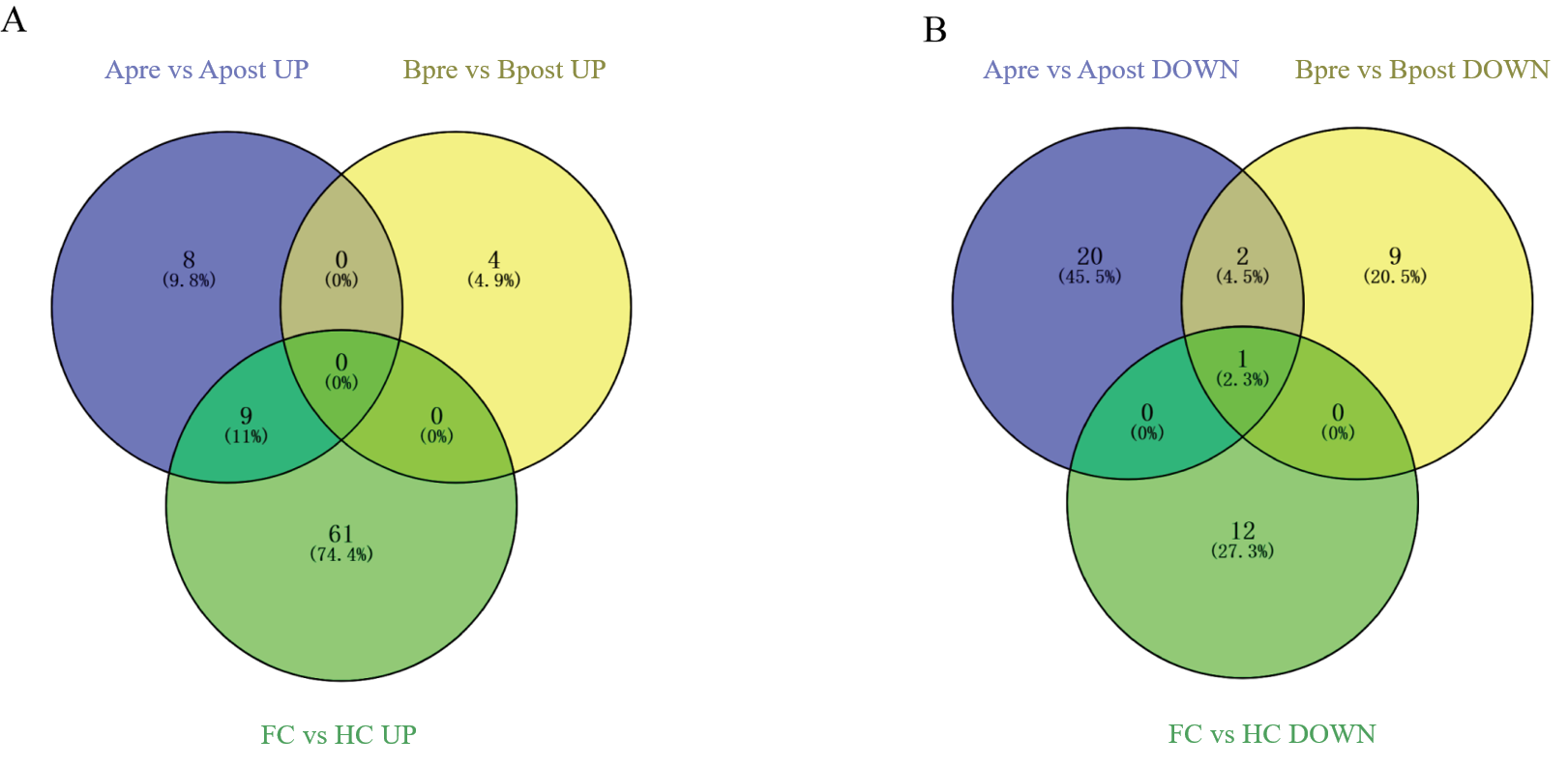 |
| --- |

Supplementary Figure S5.

| *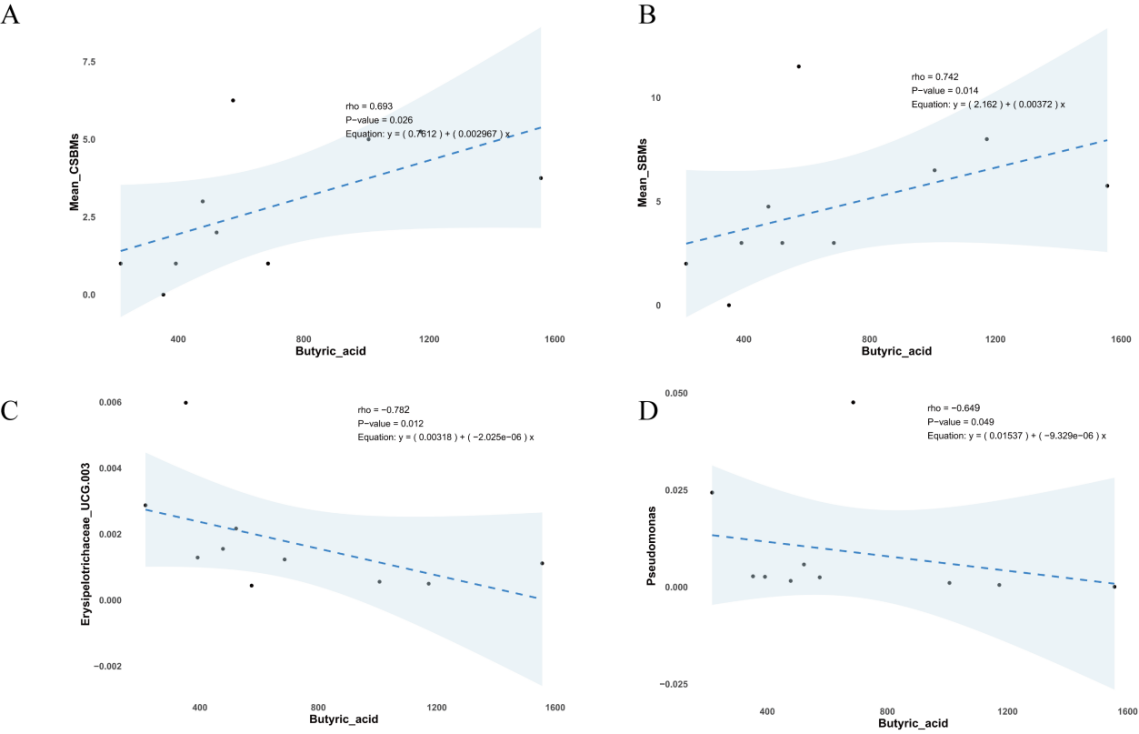* |
| --- |

Supplementary Table S1. The inclusion and exclusion criteria for FC patients

| Patients with FC |
| --- |
| **Inclusion criteria:** |
| 1. age from 18 to 60 |
| 2. meeting the Rome IV diagnostic criteria for FC: A. Two or more of the following must be conformed (more than 25% of defecations): a. exertion during evacuation; b. lumpy or hard stools; c. a sense of obstruction in the anus/rectum; d. sensation of incomplete evacuation; e. manual assistance is required for defecation; f. fewer than 3 mean weekly CSBMs; B. Loose stools rarely occur without the use of laxatives; C. Insufficient criteria for IBS; D. Criteria fulfilled for the last 3 months with symptom onset at least 6 months prior to diagnosis |
| 3. constipation for at least half a year |
| 4. no special eating habits and severe anxiety, depression, and other mental disorders (SAS or SDS standard score < 75) |
| 5. has not taken medication for constipation, including intestinal microecology and probiotics at least 2 weeks before treatment |
| 6. voluntary participation and signing informed consent |
| **Exclusion criteria:** |
| 1. irritable bowel syndrome and secondary constipation with clear etiology |
| 2. serious heart, liver, kidney damage or cognitive dysfunction, aphasia, or unable to cooperate with sample collection and treatment |
| 3. history of abdominal or anorectal surgery |
| 4. pregnant or lactating women |
| 5. other primary diseases caused by intestinal flora disorder (diabetes, obesity, migraine, etc.) |
| 6. had acupuncture treatment or participated in other clinical trials in the past three months |

Supplementary Table S2 Acupoints and non-acupoints used in the acupuncture group

| **Acupoint** | **Location** | **Non-acupoint** | **Location** |
| --- | --- | --- | --- |
| ST25 (*Tianshu*) | On the middle portion of the abdomen, 2 cun lateral to the central of the navel (needled bilaterally) | Non-acupoint 1 | On the middle of abdomen, 1 cun lateral to ST25 (*Tianshu*), the midpoint between ST25 and SP15 (*Daheng*) (needled bilaterally) |
| SP14 (*Fujie*) | On the lower abdomen, 1.3 cun below the the central of the navel and 4 cun beside the anterior midline. (needled bilaterally) | Non-acupoint 2 | On the lower abdomen, 1 cun lateral to SP14 (*Fujie*), between Stomach Meridian of Foot-Yangming and Spleen meridian of foot Taiyin (needled bilaterally) |
| ST37 (*Shangjuxu*) | On the outside of the lower leg, 6 cun below ST35 (Dubi), and on the connecting line between ST35 and ST41 (Jiexi). (needled bilaterally) | Non-acupoint 3 | On the lateral side of the lower leg, 1 cun lateral to ST37 (*Shangjuxu*), between Stomach Meridian of Foot-Yangming and Gallbladder Meridian of Foot-Shaoyang (needled bilaterally) |

Supplementary Table S3 Comparison of clinical variables at the baseline and the end of the study in different groups.

| Outcome | Acupuncture group (n=40) | Sham-acupuncture group (n=40) |
| --- | --- | --- |
| the proportion of patients with mean weekly CSBMs ≥ 3, % | | |
| Baseline | 2.50 | 7.50 |
| End of treatment | 75.00 | 7.50 |
| P value | <0.001 | 1.00 |
| mean weekly CSBMs | | |
| Baseline | 0.50 (1.00) | 1.00 (2.00) |
| End of treatment | 4.29±2.04 | 1.65±0.92 |
| P value | <0.001 | 0.009 |
| mean weekly SBMs | | |
| Baseline | 3.00 (1.00) | 3.00 (0.75) |
| End of treatment | 6.00 (2.50) | 4.75 (2.63) |
| P value | <0.001 | <0.001 |
| mean weekly straining during defecation | | |
| Baseline | 1.00 (0.67) | 1.00 (0.32) |
| End of treatment | 0.39±0.26 | 0.64±0.31 |
| P value | <0.001 | <0.001 |
| mean weekly BSFS | | |
| Baseline | 2.85±1.27 | 2.92±0.80 |
| End of treatment | 3.96±0.51 | 3.65±0.68 |
| P value | <0.001 | <0.001 |
| PAC-QOL score | | |
| Baseline | 2.51±0.66 | 2.52±0.68 |
| End of treatment | 1.44 (0.31) | 1.72 (0.72) |
| P value | <0.001 | <0.001 |
| SAS score | | |
| Baseline | 38.75 (11.25) | 41.25 (8.13) |
| End of treatment | 30.63 (8.13) | 34.38 (11.88) |
| P value | <0.001 | <0.001 |
| SDS score | | |
| Baseline | 41.72±9.04 | 44.88±9.75 |
| End of treatment | 31.25 (10.94) | 38.13 (19.69) |
| P value | <0.001 | <0.001 |

Supplementary Table S4 Specific changes in bacterial genera/species after acupuncture treatment

| Classification levels | relative abundance increase | relative abundance decrease | Elimination based on relative abundance values | Eliminated by Venne analysis |
| --- | --- | --- | --- | --- |
| genera | g_Lactobacillus  g_Agathobacter  g_Ralstonia  g_Prevotellaceae_UCG_001  g_Clostridia_vadinBB60_group  g_Methanobrevibacter | g_Parasutterella  g_Eubacterium_hallii_group  g_Phascolarctobacterium  g_Butyricimonas  g_Desulfovibrio  g_Eubacterium_coprostanoligenes_group  g_Anaerostipes  g_Intestinibacter  g_Eubacterium_ventriosum_group  g_Pseudomonas  g_Romboutsia  g_Butyricicoccus  g_Erysipelotrichaceae_UCG_003  g_UCG_005  g_Coprobacter | g_Edaphobaculum  g_Eubacterium_fissicatena_group  g_Dietzia  g_Oscillospira  g_Coprobacillus | g_CAG_352  g_Lachnospiraceae_NK4A136_group  g_Eubacterium_ruminantium_group  g_Butyrivibrio  g_Eubacterium_siraeum_group  g_UBA1819  g_Prevotellaceae_NK3B31_group  g_Collinsella  g_Ruminococcus_torques_group  g_Fusobacterium |
| species | s_Prevotellaceae_bacterium  s_Eubacterium_siraeum | s_Bacteroides_caccae  s_Parabacteroides_goldsteinii  s_Dialister_sp_  s_Bacteroides_uniformis  s_Alistipes_shahii | s_Faecalitalea_cylindroides | s_Bacteroides_dorei  s_Butyrivibrio_crossotus |

Supplementary Table S5 Specific changes in bacterial genera/species after sham acupuncture treatment

| Classification levels | relative abundance increase | relative abundance decrease | Eliminated by Venne analysis |
| --- | --- | --- | --- |
| genera | g_Streptococcus  g_Parasutterella  g_Veillonella  g_Erysipelotrichaceae_UCG_003 | g_Ruminococcus  g_Blautia  g_Sarcina  g_Clostridium_sensu_stricto_1 | g_Fusobacterium  g_Collinsella  g_Ruminococcus_torques_group |
| species |  | s_Blautia_faecis  s_Megamonas_funiformis  s_Fusobacterium_mortiferum  s_Ruminococcus_callidus  s_Bifidobacterium_longum |  |

Supplementary Table S6. Predictive function of gut microbiota in differential changes after acupuncture treatment.

| Taxa | avg(Apre) | sd(Apre) | avg(Apost) | sd(Apost) | p.value | q.values |
| --- | --- | --- | --- | --- | --- | --- |
| Cellular Processes; Cell Growth and Death; Cell cycle - Caulobacter | 0.005014743 | 0.000272487 | 0.00518572 | 0.000303087 | 0.025244414 | 0.877229578 |
| Genetic Information Processing; Folding, Sorting and Degradation; RNA degradation | 0.004599968 | 0.000149485 | 0.004684898 | 0.000158508 | 0.03700647 | 0.877229578 |
| Metabolism; Energy Metabolism; Photosynthesis proteins | 0.004451626 | 0.000337573 | 0.00464076 | 0.000294914 | 0.024471043 | 0.877229578 |
| Metabolism; Metabolism of Other Amino Acids; D-Alanine metabolism | 0.000967749 | 4.66E-05 | 0.000996086 | 5.86E-05 | 0.04285184 | 0.877229578 |
| Metabolism; Biosynthesis of Other Secondary Metabolites; Isoflavonoid biosynthesis | 1.67E-09 | 6.42E-09 | 2.63E-08 | 4.26E-08 | 0.003852195 | 0.877229578 |
